# Study of the Antimicrobial Activity of Tilapia Piscidin 3 (TP3) and TP4 and Their Effects on Immune Functions in Hybrid Tilapia (*Oreochromis* spp.)

**DOI:** 10.1371/journal.pone.0169678

**Published:** 2017-01-13

**Authors:** Chieh-Yu Pan, Tsung-Yu Tsai, Bor-Chyuan Su, Cho-Fat Hui, Jyh-Yih Chen

**Affiliations:** 1 Department and Graduate Institute of Aquaculture, National Kaohsiung Marine University, Kaohsiung, Taiwan, Taiwan; 2 Marine Research Station, Institute of Cellular and Organismic Biology, Academia Sinica, Jiaushi, Ilan, Taiwan; ContraFect Corporation, UNITED STATES

## Abstract

To address the growing concern over antibiotic-resistant microbial infections in aquatic animals, we tested several promising alternative agents that have emerged as new drug candidates. Specifically, the tilapia piscidins are a group of peptides that possess antimicrobial, wound-healing, and antitumor functions. In this study, we focused on tilapia piscidin 3 (TP3) and TP4, which are peptides derived from *Oreochromis niloticus*, and investigated their inhibition of acute bacterial infections by infecting hybrid tilapia (*Oreochromis* spp.) with *Vibrio vulnificus* and evaluating the protective effects of pre-treating, co-treating, and post-treating fish with TP3 and TP4. *In vivo* experiments showed that co-treatment with *V*. *vulnificus* and TP3 (20 μg/fish) or TP4 (20 μg/fish) achieved 95.3% and 88.9% survival rates, respectively, after seven days. When we co-injected TP3 or TP4 and *V*. *vulnificus* into tilapia and then re-challenged the fish with *V*. *vulnificus* after 28 days, the tilapia exhibited survival rates of 35.6% and 42.2%, respectively. Pre-treatment with TP3 (30 μg/fish) or TP4 (20 μg/fish) for 30 minutes prior to *V*. *vulnificus* infection resulted in high survival rates of 28.9% and 37.8%, respectively, while post-treatment with TP3 (20 μg/fish or 30 μg/fish) or TP4 (20 μg/fish) 30 minutes after *V*. *vulnificus* infection yielded high survival rates of 33.3% and 48.9%. In summary, pre-treating, co-treating, and post-treating fish with TP3 or TP4 all effectively decreased the number of *V*. *vulnificus* bacteria and promoted significantly lower mortality rates in tilapia. The minimum inhibitory concentrations (MICs) of TP3 and TP4 that were effective for treating fish infected with *V*. *vulnificus* were 7.8 and 62.5 μg/ml, respectively, whereas the MICs of kanamycin and ampicillin were 31.2 and 3.91 μg/ml. The antimicrobial activity of these peptides was confirmed by transmission electron microscopy (TEM) and scanning electron microscopy (SEM), both of which showed that *V*. *vulnificus* disrupted the outer membranes of cells, resulting in the loss of cell shape and integrity. We examined whether TP3 and TP4 increased the membrane permeability of *V*. *vulnificus* by measuring the fluorescence resulting from the uptake of 1-N-phenyl-naphthylamine (NPN). Treating fish with TP3 and TP4 under different pH and temperature conditions did not significantly increase MIC values, suggesting that temperature and the acid-base environment do not affect AMP function. In addition, the qPCR results showed that TP3 and TP4 influence the expression of immune-responsive genes, including interleukin (IL)-1β, IL-6, and IL-8. In this study, we demonstrate that TP3 and TP4 show potential for development as drugs to combat fish bacterial infections in aquaculture.

## Introduction

The tilapia (*Oreochromis niloticus*) antimicrobial peptide, piscidin, was discovered in 2012 and has been shown to perform a variety of biological functions. Five different tilapia piscidin cDNA clones have been isolated and named tilapia piscidin 1 (TP1), TP2, TP3, TP4, and TP5 [1]. TP3 exerts significant activity against bacteria [2,3], and TP4 exhibits cell proliferation-stimulating, wound closure-inducing, and bacterial infection-reducing activity [4–6]. The antimicrobial peptide piscidin-1 has been reported to have anti-nociceptive effects [1,7]. Treatment with piscidin-1 has been shown to suppress the inflammatory proteins COX-2 and iNOS in murine macrophage (RAW264.7) and microglial (BV2) cell lines stimulated with lipopolysaccharide (LPS), suggesting that piscidin-1 possesses immunomodulatory functions [7].

Piscidin genes and peptides have been identified in multiple fish species [8,9]. The structures and sequences of piscidin genes are conserved at their amino ends and contain more histidines, on average, than other proteins listed in antimicrobial databases [1,10]. Our previous research found five piscidins in *O*. *niloticus*, and studies to evaluate the minimal inhibitory concentrations (MICs) of these proteins have revealed that synthetic TP3 and TP4 peptides possess antimicrobial activity when added at 2.44~19.55 μM [1]. Although we previously described the antimicrobial activity of TP3 and TP4 against MRSA infections in mice with skin injuries, we have not yet determined the effects of these proteins on *Vibrio vulnificus* in fish or described their molecular and biological features.

Tilapia is one of the most cultivated fish species worldwide. In Taiwan and throughout the Asia-Pacific region, it is cultured in ponds, cement tanks, and FRP tanks. All tilapia are farmed in crowded conditions to maximize profit. This intensive cultivation has been reported to be an important contributing factor in disease outbreaks [11]. Antibiotics are extensively used as prophylactic agents in aquaculture to manage outbreaks and to reduce mortality. This practice has resulted in the emergence and spread of antibiotic-resistant bacteria in aquatic environments, human populations, and food animals [12]. Several recent articles have reviewed a variety of alternatives to the use of antibiotics, and one promising treatment is the use of antimicrobial peptides (AMPs) [13]. The medical industry is increasingly recognizing the potential benefits of using AMPs instead of antibiotics to control bacterial infections such as acne and catheter-related infections [13,14], and fish AMPs, such as TP3 and TP4, act as anti-infective agents with high potency against human pathogens [2,5,6]. However, no previous studies have adequately examined the antimicrobial activity of TP3 or TP4 against *V*. *vulnificus* infections in fish. Interestingly, TP3 and TP4 have demonstrated *in vivo* antimicrobial activity against *V*. *vulnificus* and *Streptococcus agalactiae*; for example, electrotransfer of the TP3 or TP4 gene into skeletal muscle resulted in enhanced antibacterial and immunomodulatory activity in *O*. *niloticus* [3].

The piscidins are a large family of AMPs, most of which are highly efficacious against both Gram-positive and Gram-negative bacteria. The excessive use of antibiotic agents in aquaculture and agriculture has led to the appearance of antibiotic-resistant strains of bacteria, including *Vibrio* sp. [12]. The drugs that are currently used to treat *V*. *vulnificus* infections include doxycycline, cephalosporin, fluoroquinolone, and trimethoprim-sulfamethoxazole plus aminoglycosides, but due to the emergence of these newly resistant bacteria, there is an urgent need to develop alternative biocontrol agents, such as AMPs. Recently, we demonstrated a high level of activity in a piscidin against carbapenem-resistant *Acinetobacter baumannii* and NDM-1-producing *Klebsiella pneumonia* in a mouse model of systemic septicemia [6]. In addition, TP3 and TP4 exhibited potent and broad-spectrum antimicrobial properties against these bacteria, which suggests that either may be a strong candidate for development as an antimicrobial agent for use in aquaculture. However, the efficacy of TP3 or TP4 against infections caused by *V*. *vulnificus* in tilapia remains unclear.

The aim of this study was to determine whether a combination treatment consisting of both peptide and non-peptide antibiotics would show improved antimicrobial activity over the use of the peptides alone. We applied the MIC technique to study the patterns of antimicrobial activity exhibited by TP3 and TP4 under different conditions (i.e., temperature and acid–base variations). The antimicrobial activity profiles of TP3 and TP4 have previously been characterized in mammal-specific applications; therefore, our study aims to fill gaps in our understanding of these peptides by extending their use to an aquatic environment.

## Materials and Methods

### Ethics statement

All experimental procedures involving animals were performed in accordance with the guidelines of Academia Sinica and approved by the Animal Care and Use Committee of Academia Sinica. We employed standardized procedures to euthanize the fish, which involved the use of buffered tricaine methane sulfonate (TMS, MS-222, Sigma Cat: A5040) to induce loss of consciousness and death. Briefly, a 10 g/L stock solution was first prepared and buffered using with bicarbonate (pH 7.0–7.5); we diluted 1 part stock solution in 39 parts water to make a 250 mg/ml working solution. The fish to be euthanized were placed in a transfer container containing diluted MS-222. It usually took 10–15 minutes for death to occur. The fish were considered dead after 10 minutes without breathing (lack of gill movement), and those that were confirmed dead were stored in a refrigerator. To reduce suffering and distress, the fish infected with *V*. *vulnificus* were cultured in water containing diluted MS-222 (100 mg/ml). The frequency of animal monitoring during the survival study were every 30 minutes to observe the tilapia (*Oreochromis* spp.) condition. Prior to infection and sampling the fish were anesthetized with MS-222 (100 mg/ml) and didn't induce apparent cause of death for those animals. At all sampling times, fish were euthanized using an overdose of MS-222 before collection of organ samples.

### Materials and microorganisms

The *V*. *vulnificus* cultures were grown in medium under the culture conditions described in a previous report [1]. We purchased TP3 (H-FIHHIIGGLFSVGKHIHSLIHGH-OH) and TP4 (H-FIHHIIGGLFSAGKAIHRLIRRRRR-OH) from GL Biochemistry (Shanghai, China) and used a solid-phase method to synthesize the synthetic peptides used in this study. These peptides were then purified to a >95% grade using reverse-phase high-performance liquid chromatography. All synthetic peptides were dissolved in ddH2O for all experiments.

### Determination of the antimicrobial activity of TP3 and TP4 against *Vibrio vulnificus*

We determined the MICs of TP3, TP4, kanamycin, and ampicillin against *V*. *vulnificus* using a broth microdilution method in accordance with NCCLS guidelines [15]. To measure the killing efficiencies of TP3 and TP4 against *V*. *vulnificus*, we prepared TP3 or TP4 solutions at MIC concentrations of 0.5×, 1×, and 2× and these solutions with equal volumes of *V*. *vulnificus* (1×10^6^ CFU/ml) to each well of a 96-well plate. The plates were incubated at 28°C for 0, 60, 120, 180, 360, 540, 720, or 1440 minutes, and after the incubation period, 50 μL of each bacterial solution were plated on a TCBS plate to obtain viable cell counts. Broth grown in the absence of peptides was used as a negative control. The results were determined using triplicate independent experiments.

### The effects of TP3 and TP4 on *Vibrio vulnificus* under synergistic and variable temperature, pH, and protease conditions

We used the co-treatment method to test combinations of TP3 or TP4 with ampicillin or kanamycin to identify synergistic effects. To measure the synergistic efficiency of TP3 or TP4 with ampicillin or kanamycin against *V*. *vulnificus*, we prepared TP3 or TP4 solutions at MIC concentrations of 0.5×, 0.25×, 0.125×, 0.0625×, 0.03125×, and 0.015625×, added them to equal volumes of *V*. *vulnificus* (5×10^3^ CFU/ml), and placed the resulting solutions in each well of a 96-well plate. The plates were incubated at 28°C for 720 minutes, and after the incubation period, the concentrations of the bacterial solutions were detected using a spectrometer (600-nm absorbance).

To understand the effects of pH on TP3 or TP4, the reagents were dissolved in ddH2O, 1 M HCl or 1 M NaOH to obtain solutions with different pH values. For the temperature-dependent functional study, the TP3 or TP4 was added to a solution in a water bath (Basic life, model BL3002, Taiwan) and incubated for 60 minutes at different temperatures (40, 60, 80, or 100°C). To evaluate the effects of TP3 and TP4 on pepsin and trypsin function, each peptide was incubated for 10 or 30 minutes with different concentrations of pepsin or trypsin (0.1, 1, or 10 μg/ml), and we determined the MIC values after incubating these solutions with cells at 28°C for 720 minutes, according to a previously described MIC experimental protocol [15].

### Membrane perturbation and morphological alteration assays

We used previously reported methods for the membrane perturbation assay [5]. Briefly, a single colony of *V*. *vulnificus* was cultured in TSB medium for 480 minutes at 28°C, and the adjusted culture (1×10^6^ CFU/ml) was aliquoted into different tubes with or without TP4 or TP3. After incubating the cells for 30 minutes, we added 40 μmol/L 1-N-phenylnaphthylamine (NPN) and continued to incubate the cells. Tubes containing PBS with NPN and bacterial cells with NPN served as controls. The NPN fluorescence intensity was detected using a fluorescence ELISA reader. All experiments were performed in triplicate, and the results are expressed as means with standard errors (SE).

We used transmission electron microscopy (TEM) and scanning electron microscopy (SEM) to evaluate the membrane integrity of *V*. *vulnificus* cells. *Vibrio vulnificus* cultures were grown for 480 minutes at 28°C, and we then added different concentrations of TP3 or TP4 and incubated the cells for 60 minutes. We then centrifuged the cells at 8000 x g for 5 min and washed and resuspended the cells in pre-fix buffer. The pre-fix buffer for SEM consisted of 2.5% glutaraldehyde in 0.1 M sodium cacodylate. The pre-fix buffer for TEM consisted of 4% paraformaldehyde and 2.5% glutaraldehyde in 0.1 M sodium cacodylate. After pre-fixing the cells in the buffer, we performed the following experimental steps at the ICOB core imaging facilities (http://icob.sinica.edu.tw/pubweb/image-core/) [16].

### Tilapia infection and rescue models

We obtained tilapia (*Oreochromis* spp.) from a commercial aquaculture company. The fish body lengths were 4.0±0.6 cm, and body weights were 3.1±0.3 g. The tilapia were maintained in a 2000 liter indoor aquarium at the Marine Research Station, Ilan, Taiwan. To study the efficacy with which TP3 and TP4 protect tilapia against *V*. *vulnificus*, we injected the bacteria into the abdominal cavities (peritoneum) of the tilapia using a syringe (29 Gx1/2, Terumo Medical, Elkton, MD, USA); control tilapia were injected with PBS-buffered saline. For the first trial in each group, we used 15 tilapia: (a) group 1 was injected with *V*. *vulnificus* (2×10^5^ CFU/fish) only; (b) group 2 was injected with PBS only; and (c) group 3 was injected with bacterial cells (2×10^5^ CFU/fish) combined with a mixture containing both TP3 and TP4 peptides (at 0.1, 1, 10, or 20 μg/fish) that had been incubated for 60 minutes at 28°C. Next, at 168 hours after the first injection, the tilapia that survived the first co-treatment were again injected with *V*. *vulnificus* (2×10^5^ CFU/fish). For this second challenge, we chose tilapia with similar body weights and ages that had not been previously treated with *V*. *vulnificus*, TP3 or TP4 for the *V*. *vulnificus*-only group (control group), and we recorded the survival rate over seven days. For the third trial, each group contained 15 tilapia, each of which was injected with 20, 30, or 40 μg of TP3 or TP4 followed by *V*. *vulnificus* (2×10^5^ CFU/fish) after 0.5, 1, or 2 hours. The survival rate was recorded after seven days. For the fourth trial, each group contained 15 tilapia, each of which was first injected with *V*. *vulnificus* (2×10^5^ CFU/fish) and then with 20, 30, or 40 μg of TP3 or TP4 after 0.5, 1, or 2 hours; the survival rate was recorded over seven days. Each experimental group consisted of 15 fish per treatment and three replicates (triplicate groups of 15 fish per treatment). We performed multiple group comparisons by analysis of variance (ANOVA) using SPSS software, and pairwise comparisons of groups were performed using a t-test. Significant differences were indicated as *P<0.05 and **P<0.01.

Prior to infection and sampling, the fish were anesthetized with buffered TMS (MS-222, Sigma, Cat: A5040). The experiment was conducted in strict accordance with the guidelines of the Animal Care and Use Committee of Academia Sinica and the regulations governing the use of animals in experimentation, and the protocol was approved by the Academia Sinica Animal Research Authority (permit no. 13-12-609). All efforts were made to minimize suffering and stress, both during handling and sampling. Humane endpoints were used, and any fish that showed signs of disease or abnormal behavior (lethargy, bloating, disoriented swimming) was euthanized by a quick blow to the head followed by dislocation of the cervical vertebra. At all sampling times, fish were almost completely exsanguinated during blood collection and euthanized with an overdose of MS-222 before organ samples were collected.

### Evaluation of antimicrobial activity in tilapia treated with synthesized TP3 and TP4

Three groups of fish were used for these experiments. The first group was first injected with *V*. *vulnificus* (2×10^5^ CFU/fish) followed by TP3 or TP4 peptides (20, 30, or 40 μg/fish) 0.5 hours later. A second group was first injected with TP3 or TP4 peptides (20, 30, or 40 μg/fish) and then, 0.5 hours later, with *V*. *vulnificus* (2×10^5^ CFU/fish). A third group of tilapia was simultaneously injected (co-treated) with *V*. *vulnificus* (2×10^5^ CFU/fish) and TP3 or TP4 peptides (20, 30, or 40 μg/fish). Liver samples were obtained from each group of tilapia at 3, 6, 9, 12, 24, and 48 hours after the final treatment and the tissues were homogenized in TSB medium before being spread onto TCBS plates. We incubated the plates at 28°C for 12 hours and then counted the number of bacterial colonies.

### Messenger (m)RNA isolation and real-time polymerase chain reaction (PCR) to analyze immune-related gene expression

Total RNA was isolated from the tilapia by peptide treatment before, concurrent with and after infection as described above. The groups included one that was only injected with *V*. *vulnificus* (2×10^5^ CFU/fish) and one that was only injected with peptides (20 μg/fish). Samples were collected at 3, 6, 12, 24, and 48 hours after treatment to extract RNA, and the levels of tilapia total RNA obtained from the liver and spleen were determined with comparative real-time qRT-PCR analysis of all experimental groups. Real-time qRT-PCR was used to detect the levels of IL-1β (GenBank accession no. DQ061114.1), IL-6 (GenBank accession no. XM_005478656), IL-8 (GenBank accession no. NM001279706), Mcp-8 (GenBank accession no. XM_005478749) and β-defensin (GenBank accession no. KF294753). mRNA was isolated and transcribed into complementary first strands (c) according to previously published methods for real-time comparative qPCR to obtain DNA and detect the relative expression levels of each gene [3,17]. The primers used in these experiments are shown in Table 1. All samples were examined in triplicate using a sample size of five tilapia. The reactions used to analyze gene expression consisted of 0.05 μg of total RNA. Comparative qRT-PCR Mastermix for the SYBR Green kit (Applied Biosystems, Perkin-Elmer) was used for quantification. The total reaction volume was 20 μl; the reactions were run in 96-well plates; and the program consisted of 40 cycles of 3 seconds at 95°C and 20 seconds at 60°C. We added a dissociation thermal start temperature of 6000B0030C to each experiment to analyze the melting peaks of the generated PCR products. The data from each experiment were expressed as a ratio of the specific target gene expression/β-actin RNA expression. We used t-tests to compare groups, and multiple group comparisons were tested by ANOVA in SPSS software. Differences were defined as significant at P<0.05 (*), <0.01 (**), and <0.001 (***).

**Table 1:** Primer used in this study.

Primer name	Sequence(5’→3’)	Accession
*Ef-1α(*F)	TCAACATCGTGGTCATTGG	AB075952
*Ef-1α*(R)	CTCAGCCTTCAGTTTGTCC	
*Il-1β(F)*	TCAGTTCACCAGCAGGGATG	DQ061114
*Il-1β*(R)	GACAGATAGAGGTTTGTGCC	
*Il-6*(F)	AGATGTCCACTGTCAAGCC	XM005478656
*Il-6*(R)	ACCGAGTAGATGAGCAGACC	
*Il-8*(F)	TGCCACACTGAAAAGGAC	NM001279704
*Il-8*(R)	AGTCATCTCGTGAAAGGAAC	
*β-defensin*(F)	TCGTGTGGTTGTTTTGGC	KF294753
*β-defensin*(R)	AGCCCAGAGGTCCAAAGAAC	
*Mcp-8*(F)	CGGGTTAGCTGTTGGCATTGT	XM005478749
*Mcp-8*(R)	AAGCAAGCAGAGAAAACCACTTCA	
*TP2*(F)	ATGAAGTGTGCTGCAGTATTTCTTATGCTGTCC	JX006071
*TP2*(R)	CTAGTCAAAATTAAGTCGACGAGGGGT	
*TP3*(F)	ATGAAGTGCACCATGCTGTTCCTTGTGCTGTCGATGGTT	JX006072
*TP3*(R)	CTAGTTAAAAGCAGCCCTTTCCC	
*TP4*(F)	GGTCGTCCTCATGGCTGA	JX006073
*TP4*(R)	GTCGTATGAGGCGATGGATAG	

### The peptide half-life in tilapia

The peptide half-life of TP3 and TP4 were investigated in male tilapia (*Oreochromis* hybrids, Tilapia spp., with a mean body length of 32.33±1.5 cm and a mean body weight of 1117±78.2 g) after intravenous administration of a single dose of 0.2 mg/kg of peptide. The TP3 and TP4 concentrations in serum samples were determined at 10, 30, 60, 120, 180, 240, 300, and 360 minutes for TP3 and at 10, 60, 120, 180, 240, 300, and 360 minutes after dosing for TP4. At each sample collection time point, we anesthetized three fish and collected their blood. The blood was centrifuged to obtain fish serum, which was assessed by liquid chromatography-mass spectrometry/mass spectrometry (LC-MS/MS) at Mission Biotech (Taipei, Taiwan).

## Results

### Antimicrobial activity of TP3 and TP4 against *V*. *vulnificus*

First, we determined the minimum MICs and minimum bactericidal concentrations (MBCs) of TP3 or TP4 against *V*. *vulnificus*. The antimicrobial activities of TP3 and TP4 against *V*. *vulnificus* are shown in Table 2. The MIC and MBC values for TP4 and TP3 against *V*. *vulnificus* were 7.8 μg/ml and 62.5 μg/ml, respectively, and the MIC and MBC values of ampicillin and kanamycin against *V*. *vulnificus* were 3.91 μg/ml and 31.2 μg/ml, respectively.

**Table 2:** Minimum inhibitory concentration (MIC) and minimum bactericidal concentration (MBC) of TP3, TP4, kanamycin, and ampicillin on *V*. *vulnificus*.

Antimicrobial agent	Bacteria	MIC (μg/ml)	MBC (μg/ml)
TP3	*V*. *vulnificus*	62.5	62.5
TP4		7.8	7.8
Kanamycin		31.2	31.2
Ampicillin		3.9	3.9

**Fig 1:**
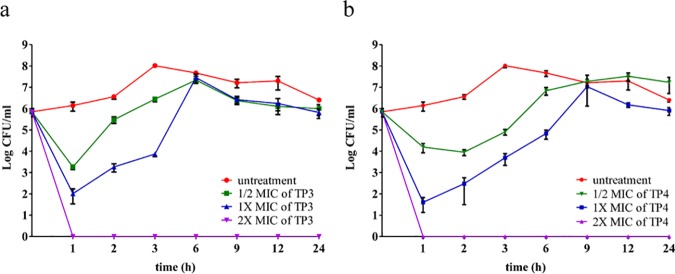
Time-kill analysis. Time-kill analysis showing the effects of (a) TP3 and (b) TP4 against *V*. *vulnificus*. Peptides at 0.5xMIC, 1xMIC, or 2xMIC were added to the bacterial cultures, which were monitored for 24 hours. Aliquots were collected at 1, 2, 3, 6, 9, 12, and 24 hours to count the bacteria. The data are the means±SE.

**Fig 2:**
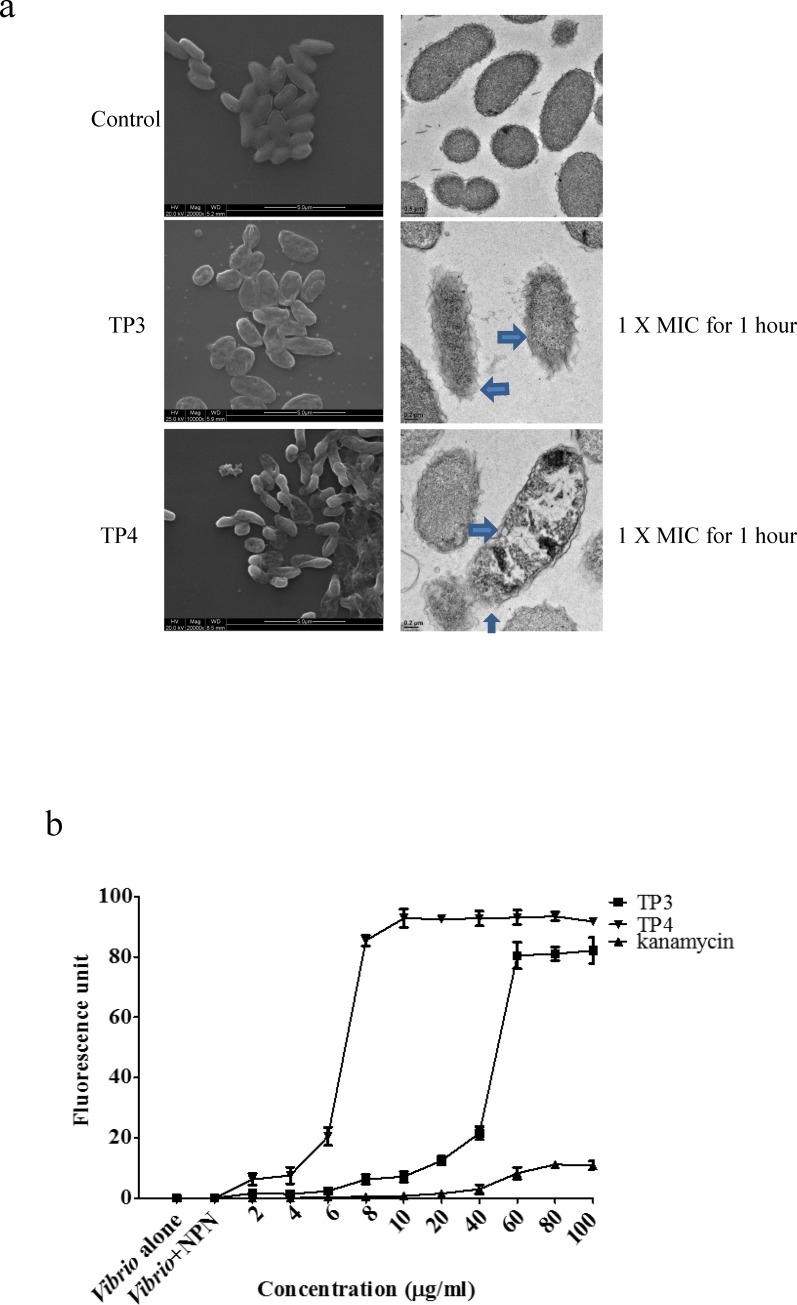
TP3 and TP4 induce membrane permeation and cause membrane disruption in V. vulnificus. (a) Treatment with TP3 or TP4 resulted in the appearance of cell injuries in *V*. *vulnificus* and the formation of electron-dense structures on the surfaces of the cells. The blue arrow indicates damage to the plasma membranes of *V*. *vulnificus* cells. The results of scanning electron microscopy (SEM) are shown on the left, and transmission electron microscopy (TEM) results are shown on the right. (b) We determined *V*. *vulnificus* membrane permeability by measuring the fluorescence resulting from the uptake of 1-N-phenyl-naphthylamine (NPN). We used cultures of *V*. *vulnificus* cells alone or *V*. *vulnificus* incubated with NPN as the control groups. Cells were treated with TP3, TP4, or kanamycin at different concentrations, and the resulting NPN fluorescence intensities were recorded to determine their correlations with membrane permeability. The data are shown as the means±SE of three independent experiments.

We observed that both TP3 and TP4 were more effective than no treatment at suppressing the growth of *V*. *vulnificus*, so we further examined the bactericidal effects of TP3 and TP4 against *V*. *vulnificus* by determining their time-kill curves. TP3 and TP4 exhibited dose-dependent bactericidal effects, which were defined as a ≥3 lg decrease in the initial inoculum (Fig 1) after 60 minutes of treatment according to the 1× and 2×MIC values. After 60 minutes, the bactericidal effect of TP3 and TP4 increased by 1× and 1/2× the MIC values, respectively (Fig 1). All bacterial strains were completely eradicated by TP3 and TP4 after 60 minutes of exposure at 2×MIC. The SEM and TEM (Fig 2A) results clearly showed differences in the morphologies of *V*. *vulnificus* that were untreated and that were treated with TP3 or TP4. The red arrow indicates that the TP3-treated *V*. *vulnificus* was reduced in size, and the blue arrowhead in the TP4 panel highlights the breaks in the plasma membrane exhibited by the *V*. *vulnificus* cells, which were also distorted. Next, we estimated the membrane integrity of *V*. *vulnificus* using 1-N-phenylnaphthylamine (NPN). The results showed that treatment with 8 μg/ml TP4 increased fluorescence, while 60 μg/ml TP3 was required to increase fluorescence, indicating that TP4 permeabilized bacterial cell membranes more effectively than TP3 (Fig 2B). Kanamycin also caused a slight increase in fluorescence intensity when applied at a higher dose of 100 μg/ml.

### Effect of pH, temperature, and proteases on antimicrobial activity against *V*. *vulnificus*

To investigate the relationship between pH and antimicrobial activity against *V*. *vulnificus*, we dissolved TP3 and TP4 in solutions with different pH values and then determined the antimicrobial activity according to the MIC and MBC values, which are presented in Table 3. TP4 and ampicillin inhibited *V*. *vulnificus* growth in pH 4 to pH 12 solutions, resulting in lower MIC and MBC values, but TP3 did not. Neither the antibiotics nor TP3 and TP4 showed any antimicrobial activity in the pH 2 solution.

**Table 3:** Analysis of the effect of pH on TP3 and TP4 antimicrobial activity.

	ampicillin		kanamycin		TP3		TP4	
	MIC (μg/ml)	MBC (μg/ml)	MIC (μg/ml)	MBC (μg/ml)	MIC (μg/ml)	MBC (μg/ml)	MIC (μg/ml)	MBC (μg/ml)
pH = 2	ND	ND	ND	ND	ND	ND	ND	ND
pH = 4	3.9	3.9	31.2	31.2	125	125	15.6	15.6
pH = 6	3.9	3.9	31.2	31.2	62.5	62.5	7.8	7.8
pH = 8	3.9	3.9	31.2	31.2	62.5	62.5	7.8	7.8
pH = 10	3.9	3.9	31.2	31.2	125	125	15.6	15.6
pH = 12	15.6	15.6	250	250	250	250	31.2	31.2
Control	3.9	3.9	31.2	31.2	62.5	62.5	7.8	7.8

Minimum inhibitory concentration (MIC) and minimum bactericidal concentration (MBC) of ampicillin, kanamycin, TP3, and TP4 on *V*. *vulnificus*. Antibiotics and peptides were dissolved in different pH solutions.

Next, we investigated the thermostability and protease stability of the investigated antimicrobial agents against *V*. *vulnificus* in the presence of pepsin and trypsin. Thermostability was measured by determining the antimicrobial activity of TP3 and TP4 after the cultures were incubated for 60 minutes at 40, 60, 80, or 100°C. We found that the MIC and MBC values were similar for TP3 and TP4, and TP3 and TP4 showed the same 1×MIC value at RT and 40°C. At 60°C, they showed the same 2×MIC value, and at 80°C and 100°C, they showed the same 4×MIC value (Table 4). The MIC and MBC values demonstrated that ampicillin is very stable from 40°C to 100°C, indicating that higher temperatures decreased the antimicrobial activity of TP3 and TP4 peptides against *V*. *vulnificus* (Table 4). The antimicrobial activity of each agent after incubation with either pepsin or trypsin for 10 or 30 minutes is summarized in Table 5. TP3 and TP4 exhibited antimicrobial activity with MIC values >500 μg/ml after incubation for 10 or 30 minutes with pepsin at a concentration of 10 μg/ml (Table 5). TP4 antimicrobial activity decreased two-fold when incubated with pepsin (1 μg/ml) for 10 minutes, and it decreased eight-fold when incubated with pepsin (0.1 μg/ml) for 30 minutes. The TP4 activity was two-fold lower when incubated with trypsin (1 μg/ml) for 10 minutes and eight-fold lower when incubated with trypsin (10 μg/ml) for 10 minutes. TP4 antimicrobial activity was reduced four-fold when incubated with trypsin (1 μg/ml) for 30 minutes and 16-fold lower when incubated with trypsin (10 μg/ml) for 30 minutes (Table 5). However, when TP3 was incubated with trypsin (10 μg/ml) for 30 minutes, we observed a loss of antimicrobial activity and MIC values >500 μg/ml. TP3 and TP4 were both more sensitive to incubation with the protease pepsin and the proteinase trypsin than they were to incubation at varying temperatures.

**Table 4:** Analysis of the effects of temperature on TP3 and TP4 antimicrobial activity.

	ampicillin		kanamycin		TP3		TP4	
	MIC (μg/ml)	MBC (μg/ml)	MIC (μg/ml)	MBC (μg/ml)	MIC (μg/ml)	MBC (μg/ml)	MIC (μg/ml)	MBC (μg/ml)
RT	3.9	3.9	31.2	31.2	62.5	62.5	7.8	7.8
40°C	3.9	3.9	31.2	31.2	125	125	15.6	15.6
60°C	3.9	3.9	31.2	31.2	125	125	15.6	15.6
80°C	3.9	3.9	31.2	31.2	250	250	31.2	31.2
100°C	3.9	3.9	62.5	62.5	250	250	31.2	31.2

Minimum inhibitory concentration (MIC) and minimum bactericidal concentration (MBC) of ampicillin, kanamycin, TP3, and TP4 at different temperatures against *V*. *vulnificus*.

**Table 5:** Analysis of protease effects on TP3 and TP4 antimicrobial activity.

Incubation time (min)	TP3			TP4		
	Pepsin (μg/ml)			Pepsin (μg/ml)		
	0.1	1	10	0.1	1	10
10	125	>500	>500	7.8	15.6	>500
30	>500	>500	>500	62.5	>500	>500
Incubation time (min)	TP3			TP4		
	Trypsin (μg/ml)			Trypsin (μg/ml)		
	0.1	1	10	0.1	1	10
10	62.5	125	>500	7.8	15.6	62.5
30	62.5	250	>500	7.8	31.2	125

Minimum inhibitory concentration (MIC) of TP3 and TP4 against *V*. *vulnificus*. Peptides were incubated with protease at different concentrations and for different exposure times.

### TP3 and TP4 exhibit synergistic activity against *V*. *vulnificus* when incubated with antibiotics

**Fig 3:**
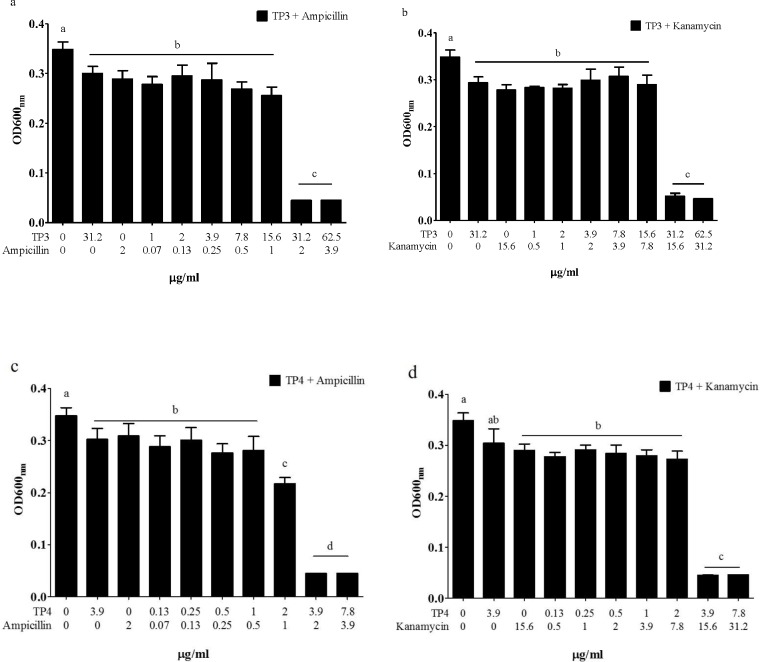
Synergistic activity of ampicillin and kanamycin in combination with TP3 or TP4. (a) The synergistic effects of TP3 and ampicillin against *V*. *vulnificus*. (b) The synergistic effects of TP3 and kanamycin against *V*. *vulnificus*. (c) The synergistic effects of TP4 and ampicillin against *V*. *vulnificus*. (d) The synergistic effects of TP4 and kanamycin against *V*. *vulnificus*. Each bar represents the mean value obtained from three experiments. Treatment results (mean±SE) marked with different numbers were significantly different (P<0.05) from each other.

Combining TP3 or TP4 with antibiotics to treat *V*. *vulnificus* infection often results in enhanced antibiotic efficacy and reduces the frequency of drug resistance. To determine the suitability of using TP3 and TP4 in combination therapies, we examined the *in vitro* dose effects of TP3 (Fig 3A) and TP4 (Fig 3B) when applied in combination with ampicillin or kanamycin. We found that both TP3 and TP4 demonstrated significant synergistic effects by which they halved the MIC of ampicillin and kanamycin (Fig 3).

### TP3 and TP4 enhance the survival of tilapia infected with *V*. *vulnificus*

**Fig 4:**
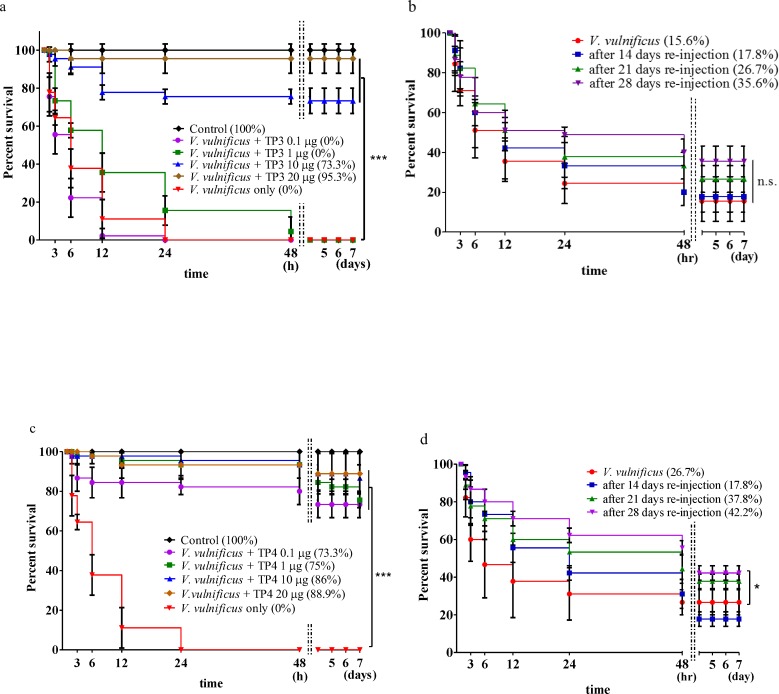
The ability of TP3 and TP4 to protect tilapia from a lethal *V*. *vulnificus* challenge in the co-treatment experiments. (a) There were six groups in this experiment: a control, either injected with only PBS or only *V*. *vulnificus* (2×10^5^ CFU/fish), or injection with a mixture of *V*. *vulnificus* (2×10^5^ CFU/fish) and TP3 peptide (0.1, 1, 10, or 20 μg/fish). The survival rates were recorded after 3, 6, 12, 24, and 48 hours for up to seven days. (b) Survival rates from experiments in which the fish that survived the above trial were re-injected and a control group was injected with a mixture of *V*. *vulnificus* (2×10^5^ CFU/fish) and TP3 peptides (20 μg/fish); the results after seven days are shown. After being re-challenged, the surviving fish from the previous experiment were continuously monitored to determine their survival rates for seven days. (c) There were six groups in this experiment: a control group, groups injected with only PBS or only *V*. *vulnificus* (2×10^5^ CFU/fish), and groups treated with a mixture of *V*. *vulnificus* (2×10^5^ CFU/fish) and TP4 peptides (0.1, 1, 10, or 20 μg/fish). The survival rates were recorded as a percentage after 3, 6, 12, 24, and 48 hours for up to seven days. (b) The survival rates of fish from the first trial that were re-challenged with a mixture of *V*. *vulnificus* (2×10^5^ CFU/fish) and TP4 peptides (20 μg/fish), which were injected simultaneously after seven days. After being re-challenged, the surviving fish were continuously monitored to determine their survival rates for seven days. Results marked with *** (P<0.001) or * (P<0.05) differed significantly among the treatments.

The antimicrobial activities of TP3 and TP4 peptides were analyzed in tilapia infected with lethal amounts of bacteria. Different amounts of *V*. *vulnificus* were injected into the abdominal cavities of the fish, and survival rates were recorded as cumulative percentages (S1 Fig). To determine whether TP3 and TP4 had any toxic effects on the tilapia, we injected different doses of TP3 and TP4 (0.1, 1, 10, 20, 30, 40, or 50 μg/fish) into the abdominal cavity and recorded the cumulative survival percentage of the fish (S2 Fig). We also co-treated tilapia with different doses (0.1, 1, 10, 20 μg/fish) of TP3 or TP4 combined with 2x10^4^ CFU of *V*. *vulnificus* to evaluate tilapia survival following treatment. When co-treated by injection with TP3 doses of 0.1, 1, 10, and 20 μg/fish, the fish achieved survival rates of 0%, 0%, 73.3%, and 95.3%, respectively (Fig 4A); only injection with 2x10^4^ CFU *V*. *vulnificus* resulted in a 0% survival rate. After 14, 21, or 28 days following the first co-treatment with TP3, the tilapia that survived in the 20 μg/fish TP3 experimental group were injected with a second dose of 2x10^4^ CFU of *V*. *vulnificus*, and the survival rates were measured for another seven days. The results showed that these second injections of 2x10^4^ CFU of *V*. *vulnificus* resulted in survival rates of 17.8%, 26.7%, and 35.6% after 14, 21, or 28 days, respectively (Fig 4B). When co-treated with doses of 0.1, 1, 10, and 20 μg/fish TP4, the fish achieved survival rates of 73.3%, 75%, 86%, and 88.9% respectively (Fig 4C). In the co-treatment experiments, at 14, 21, or 28 days after the first co-treatment with TP4, the surviving tilapia in the 20 μg/fish TP4 experimental group received a second injection of 2x10^4^ CFU of *V*. *vulnificus*, and the survival rates were measured for another seven days. The results showed that after 14, 21, or 28 days, the survival rates were 17.8%, 37.8%, and 42.2%, respectively (Fig 4D).

**Fig 5:**
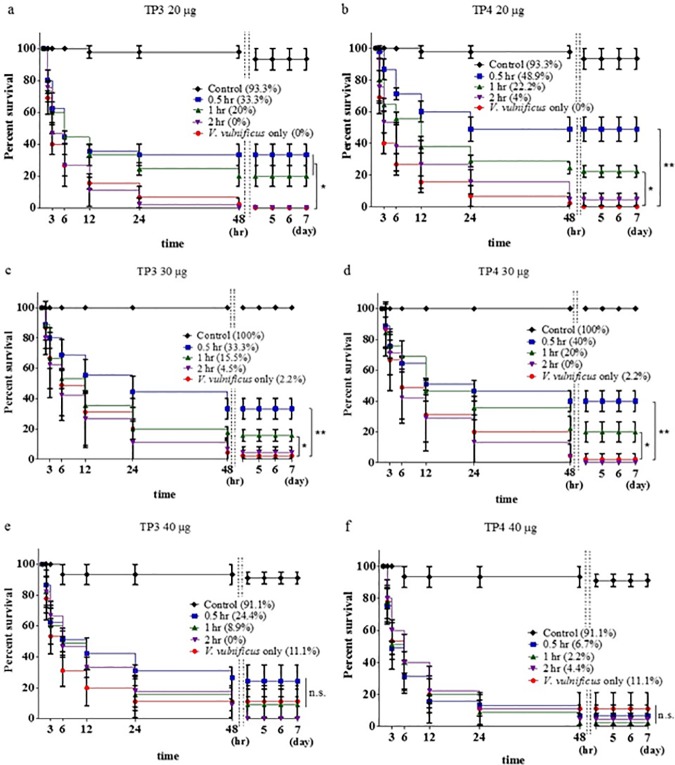
Time-dependent effects of pre-treatment with *V*. *vulnificus* followed by injection with TP3 or TP4. (a) Tilapia were infected with *V*. *vulnificus* (2×10^5^ CFU/fish) and then injected with TP3 (20 μg/fish) or PBS (control) after 0.5, 1, or 2 hours. Another control group was injected with only *V*. *vulnificus* (2×10^5^ CFU/fish). (b) Tilapia were first infected with *V*. *vulnificus* (2×10^5^ CFU/fish) and then injected with TP4 (20 μg/fish) or PBS (control) after 0.5, 1, or 2 hours, and another control group was injected with only *V*. *vulnificus* (2×10^5^ CFU/fish). (c) The same experimental method described in (a) was used, but the fish were treated with TP3 (30 μg/fish). (d) The same experimental method described in (b) was used, but the fish were treated with TP4 (30 μg/fish). (e) The same experimental method described in (a) was used, but the fish were treated with TP3 (40 μg/fish). (f) The same experimental method described in (b) was used, but the fish were treated with TP4 (40 μg/fish). ns, not significantly different. Results marked with ** (P<0.01) or * (P<0.05) differed significantly among the treatment groups.

In this study, we treated tilapia with *V*. *vulnificus* (2x10^4^ CFU/fish) for 0.5, 1, or 2 hours before injecting the fish with 20 μg, 30 μg, or 40 μg of TP3 or TP4 to evaluate mortality. When the fish were pre-treated with an injection of *V*. *vulnificus* (2x10^4^ CFU/fish) followed by an injection of TP3 (20 μg/fish) after 0.5, 1, or 2 hours, the survival rates were 33.3%, 20%, and 0%, respectively, at the end of the seven-day experimental period (Fig 5A). Injecting fish with *V*. *vulnificus* (2x10^4^ CFU/fish) and then TP3 (30 μg/fish) resulted in survival rates of 33.3%, 15.5%, and 4.5% after 0.5, 1, or 2 hours, respectively, at the end of the seven-day experimental period (Fig 5C). When the fish were pre-treated with an injection of *V*. *vulnificus* (2x10^4^ CFU/fish) followed by an injection of TP3 (40 μg/fish) after 0.5, 1, or 2 hours, the survival rates were 24.4%, 8.9%, and 0%, respectively, at the end of the seven-day experimental period (Fig 5E). To determine the curative potential of TP4, we first injected tilapia with *V*. *vulnificus* (2x10^4^ CFU/fish) and then injected the fish with TP4 at 20 μg/fish, 30 μg/fish, or 40 μg/fish after 0.5, 1, or 2 hours. Pre-treating the fish in this way resulted in survival rates of 48.9%, 22.2%, and 4%, respectively, at the end of the seven-day experimental period (Fig 5B). When pre-treated with an injection of *V*. *vulnificus* (2x10^4^ CFU/fish) followed by an injection of TP4 at a dose of 30 μg/fish after 0.5, 1, or 2 hours, the survival rates were 40%, 20%, and 0%, respectively, at the end of the seven-day experimental period (Fig 5D). When pre-treated with an injection of *V*. *vulnificus* (2x10^4^ CFU/fish) followed by an injection of TP4 (30 μg/fish) after 0.5, 1, or 2 hours, the survival rates were 2.2%, 4.4%, and 6.7%, respectively, at the end of the seven-day experimental period (Fig 5F).

**Fig 6:**
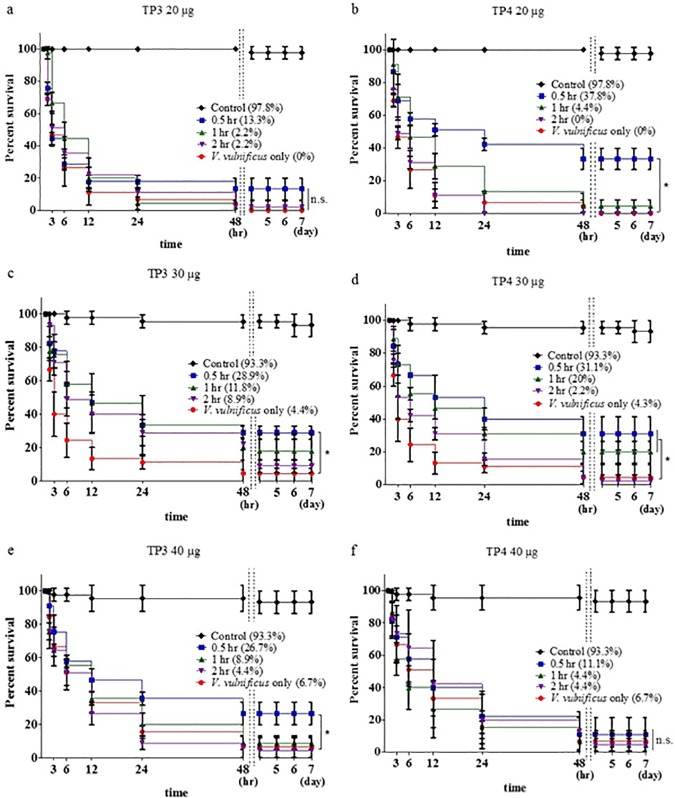
Time-dependent effects of pre-treatment with TP3 or TP4 followed by infection with *V*. *vulnificus*. (a) The tilapia were first treated with TP3 (20 μg/fish) and then with *V*. *vulnificus* (2×10^5^ CFU/fish) or PBS (control) after 0.5, 1, or 2 hours. One group was injected with only *V*. *vulnificus* (2×10^5^ CFU/fish) as a control. (b) Tilapia were first treated with TP4 (20 μg/fish) and then injected with *V*. *vulnificus* (2×10^5^ CFU/fish) or PBS (control) after 0.5, 1, or 2 hours; one group was injected with only *V*. *vulnificus* (2×10^5^ CFU/fish) as a control. (c) The same experimental methods described in (a) were used, but the fish were treated with TP3 (30 μg/fish). (d) The same experimental methods described in (b) were used, but the fish were treated with TP4 (30 μg/fish). (e) The same experimental methods described in (a) were used, but the fish were treated with TP3 (40 μg/fish). (f) The same experimental methods described in (b) were used, but the fish were treated with TP4 (40 μg/fish). ns, not significantly different. Results marked with * (P<0.05) differed significantly among the treatments.

To study the bactericidal effects of TP3 and TP4, we monitored their impact on the survival of tilapia infected with *V*. *vulnificus*, and the results showed that after treatment with TP3 (20 μg), higher survival rates of 13.3%, 2.2%, and 2.2% were achieved after 0.5, 1, or 2 hours, respectively, than those observed in fish injected with *V*. *vulnificus* alone. The survival rates were evaluated after 7 days (Fig 6A). Treating the fish with TP3 (30 μg) resulted in higher survival rates of 28.9%, 11.8%, and 8.9% after 0.5, 1, or 2 hours, respectively, than were observed in fish injected with *V*. *vulnificus* alone (Fig 6C). Treatment with TP3 (40 μg) resulted in higher survival rates of 26.7%, 8.9%, and 4.4% after 0.5, 1, or 2 hours, respectively, than those in fish injected with *V*. *vulnificus* alone (Fig 6E). However, at 7 days after infection with *V*. *vulnificus*, the survival rates were 37.8%, 4.4%, and 0%, respectively, in fish that had been pre-treated with 20 μg/fish TP4 (Fig 6B); 31.1%, 20%, and 2.2% in fish pre-treated with 30 μg/fish TP4 (Fig 6D); and 11.1%, 4.4%, and 4.4% in fish pre-treated with 40 μg/fish TP4 (Fig 6F). Our results indicate that TP3 or TP4 have strong antiseptic activities in tilapia, suggesting that TP3 or TP4 play significant roles in protecting tilapia against *V*. *vulnificus* infections that normally lead to septic death.

### *In vivo* bactericidal activities of TP3 and TP4 in tilapia livers

**Fig 7:**
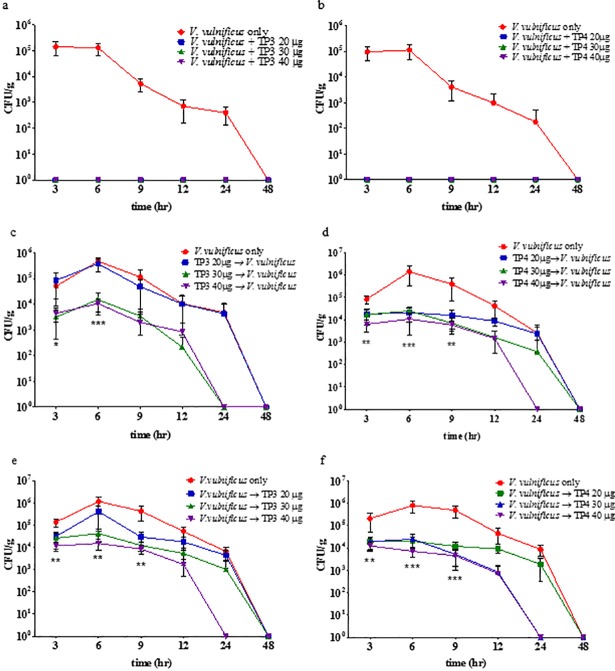
Analysis of in vivo bacteriostatic properties. The in vivo bacteriostatic properties of animals injected with TP3 or TP4 in a tilapia model in which peritonitis is induced by an intraperitoneal injection of *V*. *vulnificus* (2×10^5^ CFU/fish). (a) Tilapia were simultaneously injected with TP3 (20 μg/fish, 30 μg/fish, or 40 μg/fish) and *V*. *vulnificus*. (b) Tilapia were simultaneously injected with TP4 (20 μg/fish, 30 μg/fish, or 40 μg/fish) and *V*. *vulnificus*. (c) Tilapia were first treated with TP3 (20 μg/fish, 30 μg/fish, or 40 μg/fish) and then with *V*. *vulnificus* after 30 minutes. (d) Fish were first treated with TP4 (20 μg/fish, 30 μg/fish, or 40 μg/fish) and then infected with *V*. *vulnificus* after 30 minutes. (e) Fish were infected with *V*. *vulnificus* first and then, after 30 minutes, injected with TP3 (20 μg/fish, 30 μg/fish, or 40 μg/fish). (f) Fish were infected with *V*. *vulnificus* first and then, after 30 minutes, injected with TP4 (20 μg/fish, 30 μg/fish, or 40 μg/fish). Livers were obtained from the tilapia at 3, 6, 9, 12, 24, and 48 hours after treatment. Colony counts are shown as the mean±SE. Significant treatment differences (** P<0.01; *** P<0.001) were determined by group comparisons.

We evaluated the *in vivo* antimicrobial activity of TP3 and TP4 in tilapia by inducing *V*. *vulnificus* infections and then treating the fish with the peptides. Treating fish infected with *V*. *vulnificus* with TP3 or TP4, either as a co-treatment, pre-treatment, or post-treatment, resulted in a comparable reduction in the bacterial load in the liver. Treatment with TP3 resulted in a significantly lower *V*. *vulnificus* bacterial load in the liver than was observed in the livers of fish that were infected but not treated (Fig 7A, 7C and 7E). The TP4 treatment also significantly reduced the *V*. *vulnificus* bacterial load in the liver compared to that observed in the livers of fish that were infected but untreated (Fig 7B, 7D and 7F). These results indicate that TP3 and TP4 efficiently control *V*. *vulnificus* in the livers of infected tilapia.

### Analysis of differentially expressed genes by qPCR

To determine whether co-treatment, post-treatment or pre-treatment with synthesized TP3 or TP4 peptides to combat bacterial infection could modulate immune-related gene expression profiles in tilapia, we used *V*. *vulnificus* to stimulate immune responses and quantified the transcriptional levels of immune-related genes in tilapia livers and spleens. As shown in S3 Fig, significantly higher levels of IL-1β, IL-6, IL-8, Mcp-8, and β-defensin gene expression were observed in the liver tissues of the TP3 pre-treatment and post-treatment groups (S3 Fig) three hours after infection with *V*. *vulnificus*. TP3 pre-treatment and post-treatment resulted in a significant increase in the gene expression levels of IL-1β, IL-6, IL-8, and β-defensin in spleen tissues three hours after infection (S3B Fig). The observed differences in the gene expression levels of IL-1β, IL-6, IL-8, Mcp-8, and β-defensin between the fish treated with TP3 alone and those in the co-treatment groups were significant in all of the test groups. In liver tissues, higher levels of IL-1β, IL-6, and IL-8 were induced by treatment with *V*. *vulnificus* alone after 3, 6, 12, 24, and 48 hours (S3C Fig), but infection in fish treated with TP4 or co-injected with TP4+*V*. *vulnificus* did not produce significantly different results from those observed in the *V*. *vulnificus*-only group after either 24 or 48 hours, which was similar to the effects observed for IL-6, IL-8, and β-defensin (S3C Fig). The expression levels of IL-1β, IL-6, IL-8, and Mcp-8 were lower in the spleens of fish in the TP4 or TP4+*V*. *vulnificus* (co-) injected groups 12 hours after treatment (S3D Fig). All other genes were either up- or down-regulated in the *V*. *vulnificus*-injected group, but their expression levels were significantly lower in the liver and spleen tissues of the fish in the TP4-injected and TP3-injected groups (S3 Fig).

### Analysis of the TP3 and TP4 peptide half-life in tilapia serum

To better understand the biological half-life of TP3 and TP4, we obtained tilapia serum and determined the concentration of TP3 or TP4 by LC-MS/MS (Mission Biotech, Taiwan). The half-life of the injected TP3 peptide was 120–180 minutes, and that of TP4 was 60 minutes, indicating that TP3 or TP4 may be active in the early stages of infection, so more bacteria were eliminated with higher doses of TP3 (S4A Fig) or TP4 (S4B Fig) in solution in the co-treatment experiment.

## Discussion

Interest in TP3 and TP4 has grown significantly in recent years because the functions of these factors in bacterial infection therapies have been demonstrated [3–5]. Currently, bacterial resistance to conventional antibiotics has reached hazardous levels and may represent an impending return to the pre-antibiotic era [18]. Previous publications have reported a lack of regulation and enforcement of antibiotic use among aquaculture farmers in developing countries [11]. The misuse of antibiotics as prophylactic agents to prevent disease has given rise to the development of antibiotic resistance [19]. TP3 and TP4 exert biological effects against microbes, which indicates that they are potent and promising antimicrobial agents with a broad spectrum of activities [1]. In addition, TP3 and TP4 are also involved in modulating immune and inflammatory responses [3]. Excessive antibiotic use is a major cause of imbalances in the normal distributions of bacterial species in aquatic environments. Moreover, *V*. *vulnificus* is currently developing resistance to many clinically important antibiotics. Because *V*. *vulnificus* infections progress rapidly, several therapeutic methods are currently available to treat *V*. *vulnificus* infections in humans, such as a combination of tetracycline and cephalosporin or monotherapy with fluoroquinolone, as recommended by the CDC [20]. *Vibrio vulnificus* bacteria are etiological agents that reside in warm water and cause vibriosis in tilapia, eels and other teleosts in fish farms. Recently, the detection of antimicrobial resistance in *Vibrio* isolates in aquaculture environments has suggested that resistant *Vibrio* strains may pervade the food chain. They may therefore eventually reach humans and present a risk to public health [21].

To understand how TP3 and TP4 could potentially be used as alternatives to conventional antibiotics, we studied their biochemical stability under different stimulated *in vivo* conditions, including exposure to different pH, temperature, and protease-rich environments. Recently, we reported on the use of TP4 against *Helicobacter pylori* infections *in vivo* and *in vitro* [5]. The results suggested that TP4 possesses antimicrobial activity at lower pH values. In the present study, we confirmed that TP3 and TP4 could exert potent bactericidal activity against *V*. *vulnificus* across a range of different pH values, and an important result was that TP3 and TP4 were observed in the buffered sample at pH 7 (control), confirming that the research results for the pH control from the previous study remain valid. At pH 4, pH 10, and pH 12, the TP3 and TP4 peptides assumed an entirely unknown structure that could not be predicted, which may have caused them to lose cationic features that resulted in an increase in MIC values. The unbuffered PGLa samples were significantly acidic and at the edge of the acceptable pH window [22]. These results probably also apply to most other cationic membrane-active peptides, including TP3 and TP4, suggesting that these peptides merit further biophysical analysis. Therefore, the usefulness of applying TP3 or TP4 as a supplement to fish fodder may be limited by their specific properties, such as temperature tolerance and protease effects. Our results showed that TP3 and TP4 remained stable at temperatures varying from room temperature to as high as 100°C and that 16 hours of incubation with 31.2 μg/ml of TP4 or 250 μg/ml of TP3 was sufficient to eliminate *V*. *vulnificus*. These results indicate that TP3 and TP4 can remain stable throughout the fish fodder manufacturing process, thereby ensuring the antimicrobial activity of the product. Another study showed that an antimicrobial protein derived from *Bacillus subtilis* FB123, named BSAMP, was highly thermostable after treatment at 100°C; its antimicrobial activity did not change [23]. Northeast red beans produce thermostable and pH-stable defensin-like peptides that fully maintain their antimicrobial activity at temperatures as high as 100°C and at pH values ranging from 0 to 12 [24]. The apparent stability of these peptides following exposure to heat or different pH values could potentially be attributed to their ability to promote *in vitro* aggregation of the acidic phospholipid dimyristoylphosphatidylserine or interactions that result in a reduction to their cationic charges [25].

Here, we used NPN uptake assays and SEM to demonstrate that TP3 or TP4 can disrupt membranes at low MIC values. Based on the SEM results, we propose that TP3 or TP4 destabilize the cell membranes of *V*. *vulnificus* by promoting vesicular budding, blebbing, and shrinking. We previously reported that the antimicrobial activity of TP4 is correlated with the induction of membrane micelle formation, which leads to membrane depolarization and the extravasation of cellular constituents [5]. We devised this study to evaluate the synergistic effects among antibiotics and TP3 or TP4 when applied as treatments for *V*. *vulnificus* infections. We compared the results to those obtained using a commonly applied *V*. *vulnificus* antibiotic treatment regimen (ampicillin and kanamycin). The greatest benefit of integrating the use of TP3 or TP4 with antibiotics is the decrease in the required therapeutic dosage. Lower doses reduce potential side effects, which is an important factor during drug development. We observed significant improvements in MIC values when we used TP3 and TP4 against *V*. *vulnificus*, but the synergy between TP3/4 and antibiotics resulted in dramatic decreases in the MICs of both TP3 (from 31.2 to 62.5 μg/ml) and TP4 (from 3.91 to 7.81 μg/ml) against *V*. *vulnificus*. In addition, when combined with TP3 or TP4, the antibiotics induced a two-fold decrease in MIC values. The observation that both ampicillin and kanamycin interacted synergistically with TP3 or TP4 suggests the involvement of different mechanisms between these peptides and the applied antibiotics [26,27]. One mechanism of interest in relation to these alternative factors may be that they increase membrane perturbations or pore formation on bacterial cell walls, both of which can enhance the uptake of antibiotics and thereby increase their antibacterial effects [28]. When we used TP3 and TP4 at 1xMIC, the *V*. *vulnificus* CFUs recovered after approximately 6 and 9 hours, respectively, which may due to antimicrobial peptide (AMP) degradation by the bacteria. Because the 1xMIC only presented the AMP minimum inhibitory concentration, bacterial growth and recovery will occur after exhaustion of the AMP molecules. However, this must be verified for both TP3 and TP4 in future experiments.

Prophylactic or post-treatment administration of TP3 or TP4 protected tilapia against serious sepsis when they were infected with *V*. *vulnificus*. Septic shock was induced by intraperitoneal administration of *V*. *vulnificus* using experimental methods that have been described in several animal models [29,30]. The percent survival after injection of only TP3 at 20, 30, 40, 50 μg/fish was 86.7%, 73.3%, 66.7%, and 56.7%, respectively. The percent survival with TP4 alone at 20, 30, 40, 50 μg/fish was 70.0%, 56.7%, 63.3%, and 30.0%, respectively. Figs 5 and 6 show that the percent survival with either TP3 or TP4 treatment dosages from 20 μg to 40 μg were lower than those with only TP3 (or TP4) treatment at 20, 30, 40, 50 μg/fish (S2 Fig), suggesting that fish death may have been due to bacterial infection and inherent peptide toxicity. Similar to epinecidin-1, we found that TP3 and TP4 rapidly killed *V*. *vulnificus* and reduced the number of its bacterial colonies in tilapia. Here, we provide both *in vitro* and *in vivo* experimental confirmation that TP3 and TP4 possess potent antimicrobial activity against *V*. *vulnificus* and that TP3 and TP4 permeated *V*. *vulnificus* membranes and balanced the host immune response in a tilapia model of *V*. *vulnificus* infection. Treating the fish with TP3 and TP4 resulted in 100% bacterial clearance of *V*. *vulnificus* from the tilapia after 48 hours. Treatment with TP4 has also been previously reported to reduce bacterial (*Helicobacter pylori*) loads in mice [5]. There are several ways to stabilize TP3 or TP4 *in vivo*, such as using liposome-packaged peptides, nanoparticles, or D-form peptides [31,32], but these experimental methods are not suitable for aquacultural applications due to cost issues. Therefore, additional analyses are required to determine the best strategy for peptide stabilization and reducing the costs associated with the manufacture of TP3 or TP4 in fish fodder. The peptide half-life experimental results in tilapia suggested the concentration of TP3 or TP4 peptides that could effectively inhibit *V*. *vulnificus* growth either *in vitro* or *in vivo*, and they were consistent with previously published data for other peptide treatments such as epinecidin-1 [33]. Taken together, these findings highlight the usefulness of TP3 and TP4 for the treatment of *V*. *vulnificus*-infected tilapia.

To better understand the responses of tilapia to *V*. *vulnificus* infection after administration of TP3 or TP4 via different treatment methods, we analyzed the qPCR experimental results, which are presented in the supporting information. These results indicated that the TP2, TP3, TP4 or β-defensin antimicrobial peptides were down-regulated in the liver in response to either TP3 or TP4 treatment alone or in co-treatment groups 3 hours after treatment. These results suggest that immunomodulation of antimicrobial peptide (AMP)-responsive genes by TP3 or TP4 in the early stages of infection did not enhance AMP gene expression, and this may be a consequence of the TP3 (or TP4) co-treatment with *V*. *vulnificus*, in which TP3 (or TP4) destroyed the *V*. *vulnificus* membrane and reduced the disease severity after bacterial infection. These results reveal a similar function in tilapia (e.g., antimicrobial peptide) as found through parallel observations in zebrafish [34]. In particular, immune-related genes, such as IL-1β, IL-6, IL-8, and MCP-8, were down-regulation by TP3 or TP4 co-treatment or by TP3 or TP4 treatment alone, suggesting that TP3 or TP4 are potent modulators of immune-related gene responses [34]. The *V*. *vulnificus* VvpE stimulates IL-1β production via hypomethylation of the IL-1β promoter and NF-κB activation in the intestinal epithelial cells [35], but whether TP3 or TP4 are involved in IL-1β promoter and NF-κB activation in tilapia after *V*. *vulnificus* infection requires further investigation. Tilapia infected with *V*. *vulnificus* exhibit severe inflammatory responses characterized by the regulation of proinflammatory cytokines such as mast cell protease 8 (MCP-8), increasing the inflammation caused by sepsis. Mast cell proteases are major components of the secretory granules of mature mast cells, but whether MCP-8 regulates the responses to *V*. *vulnificus* infection, as well as the actual mechanism by which *V*. *vulnificus* initiates production of these MCP-8-modulated proinflammatory cytokines, remain unclear. We suspect that TP3 (or TP4) may affect tilapia MCP-8 gene expression after *V*. *vulnificus* infection and the mast cell protease-dependent proteolysis in tilapia defense responses to invading pathogens [36,37].

Overall, TP3 and TP4 are interesting therapeutic candidates that could influence immune-related gene expression in fish. It is now well established that TP3 and TP4 act as antimicrobial and immunomodulatory factors by disrupting membrane structures. A few AMPs, such as Pexiganan (MSI-78), Iseganan (IB-367) and Omiganan (MBI 226, CLS001), have advanced to phase III trials that will provide new opportunities for treating these types of infections [38]. New types of therapeutic interventions involving intravenous or topical cream applications of TP3 and TP4 merit further investigation.

## Supporting Information

S1 Fig Determination of bacterial numbers and survival rates. (a) We monitored bacterial numbers and (b) survival rates after tilapia were infected with different amounts of *V*. *vulnificus*. Data were recorded after 3 hours, 6 hours, 12 hours, 24 hours, 48 hours, 4 days, 5 days, 6 days and 7 days.
(PDF)

S2 Fig Determination of tilapia survival rates seven days after injection with different concentrations of TP3 or TP4. (a) The survival rates of tilapia injected with TP3 at a dose of 0.1 μg/fish, 1 μg/fish, or 10 μg/fish. (b) The percentage of tilapia surviving after injection with TP4 at 0.1 μg/fish, 1 μg/fish, or 10 μg/fish. (c) The percentage of tilapia surviving after injection with TP3 at 20 μg/fish, 30 μg/fish, 40 μg/fish, or 50 μg/fish. (d) The percentage of tilapia surviving after injection with TP4 at 20 μg/fish, 30 μg/fish, 40 μg/fish, or 50 μg/fish. The control group (control) was injected with PBS alone.
(PDF)

S3 Fig Differential expression of immune-related genes in the livers and spleens of tilapia after co-, pre-, or post-treatment with TP3 or TP4. (a) TP3-treated livers, (b) TP3-treated spleens, (c) TP4-treated livers, and (d) TP4-treated spleens. Comparative RT-PCR analysis of the mRNA gene expression levels of immune-related genes. The evaluated genes are shown in Table 1 before and after different experimental conditions, as described in Fig 7. Each bar represents the mean value of three experiments including the SE. The results (mean±SE) marked with asterisks were significantly different (* P<0.05, ** P<0.01, *** P<0.001) among the treatments: *Vibrio*, injected with *V*. *vulnificus* alone; TP4, injected with TP4 alone; TP3, injected with TP3 alone; co, co-treatment with AMP and *V*. *vulnificus*; pre, prior treatment with AMP followed by infection with V. vulnificus; post, infection with *V*. *vulnificus* followed by treatment with AMP.
(PDF)

S4 Fig The tilapia piscidin 3 and tilapia piscidin 4 peptide half-life in tilapia. (a) Pharmacokinetic curve for tilapia after injection of a single dose (0.2 mg/kg; using three fish at each time point) of TP3 and (b) TP4. Serum concentrations were determined by intravenous sampling at each time point, and piscidin concentrations were determined by liquid chromatography-mass spectrometry/mass spectrometry (LC-MS/MS).
(PDF)
