# Supplementary figures and images for: Study of the Antimicrobial Activity of Tilapia Piscidin 3 (TP3) and TP4 and Their Effects on Immune Functions in Hybrid Tilapia (Oreochromis spp.)
Source: PLoS One. 2017 Jan 13;12(1):e0169678. doi: 10.1371/journal.pone.0169678 (PMC5234776; doi:10.1371/journal.pone.0169678)

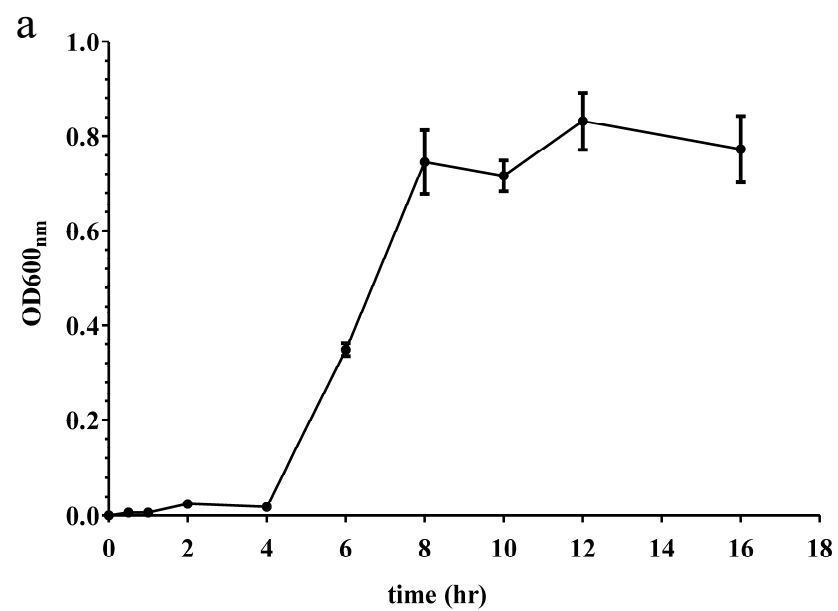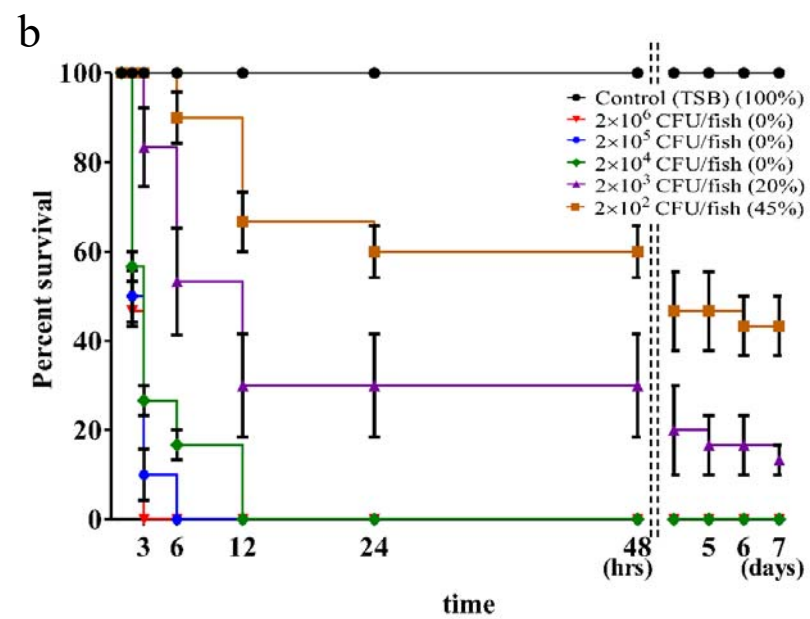

Supplement: S1 Fig — (a) We monitored bacterial numbers and (b) survival rates after tilapia were infected with different amounts of V. vulnificus. Data were recorded after 3 hours, 6 hours, 12 hours, 24 hours, 48 hours, 4 days, 5 days, 6 days and 7 days. (PDF) [file pone.0169678.s001.pdf]

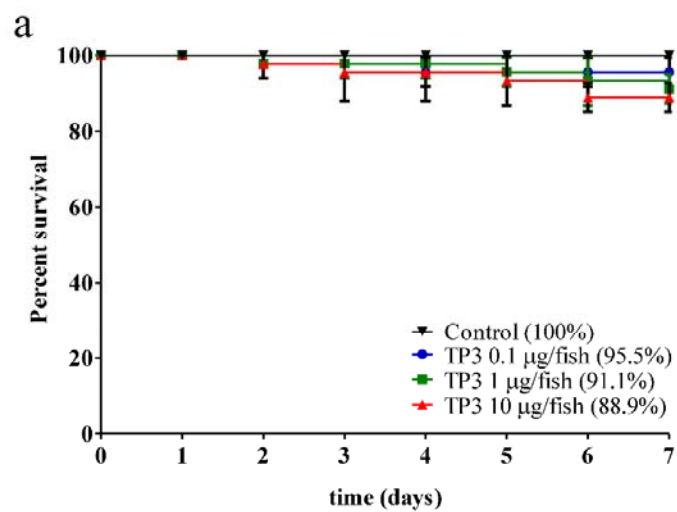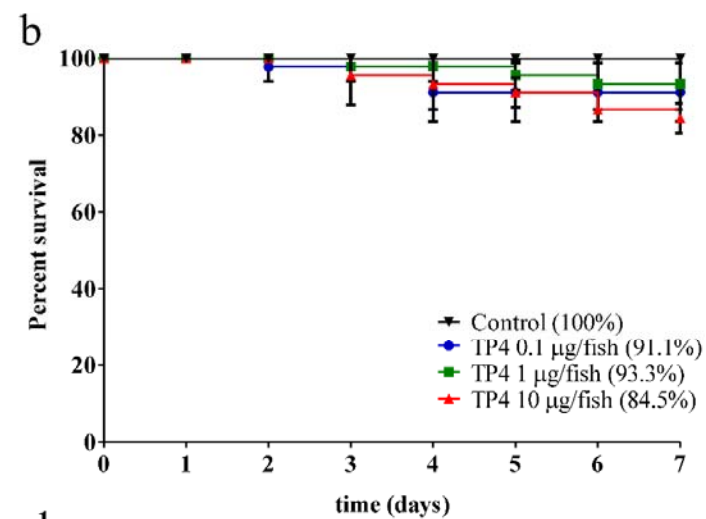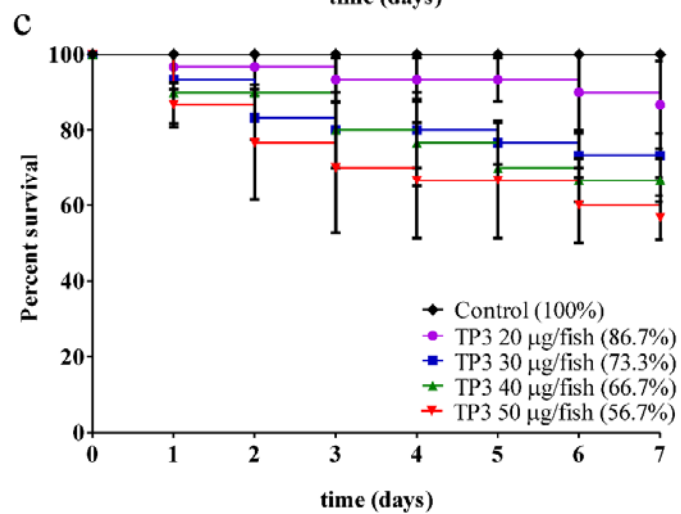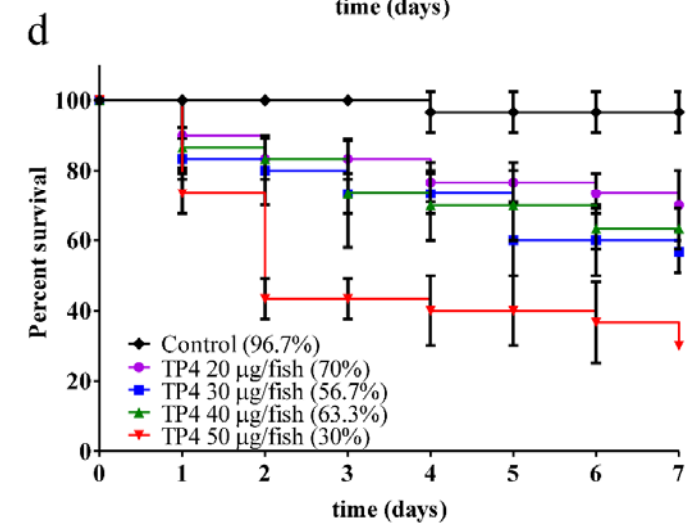

Supplement: S2 Fig — (a) The survival rates of tilapia injected with TP3 at a dose of 0.1 μg/fish, 1 μg/fish, or 10 μg/fish. (b) The percentage of tilapia surviving after injection with TP4 at 0.1 μg/fish, 1 μg/fish, or 10 μg/fish. (c) The percentage of tilapia surviving after injection with TP3 at 20 μg/fish, 30 μg/fish, 40 μg/fish, or 50 μg/fish. (d) The percentage of tilapia surviving after injection with TP4 at 20 μg/fish, 30 μg/fish, 40 μg/fish, or 50 μg/fish. The control group (control) was injected with PBS alone. (PDF) [file pone.0169678.s002.pdf]

(3a) TP3 treatment of liver

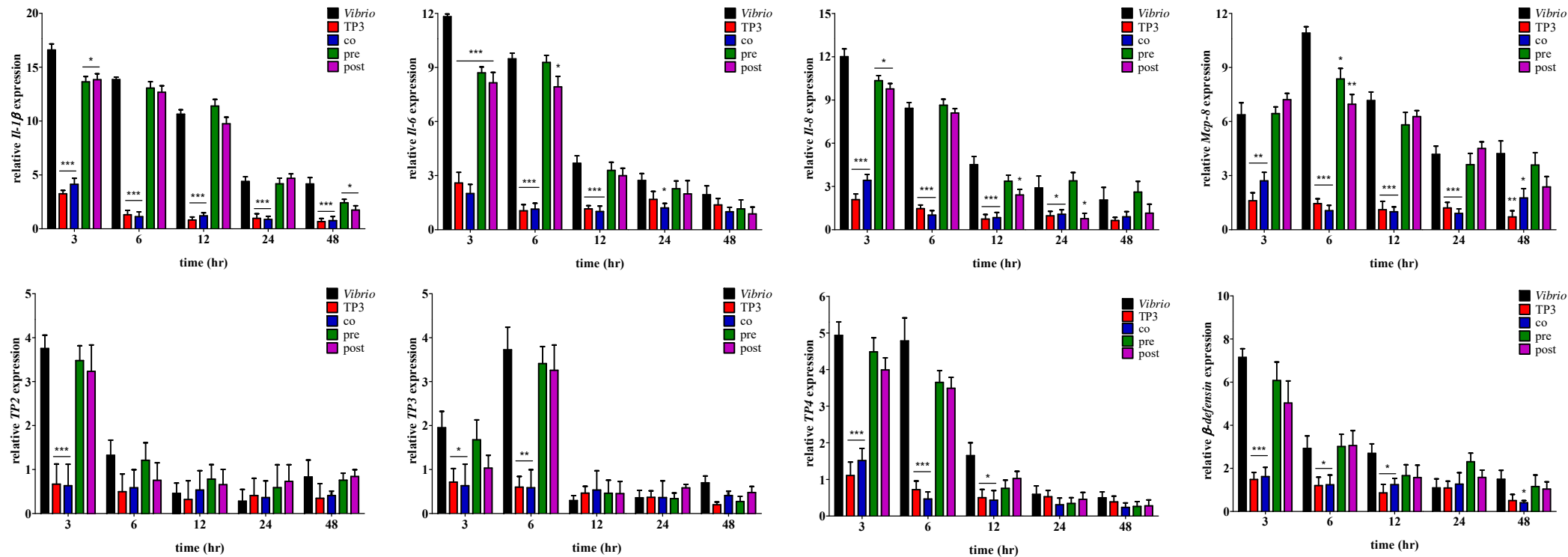

(3b) TP3 treatment of spleen

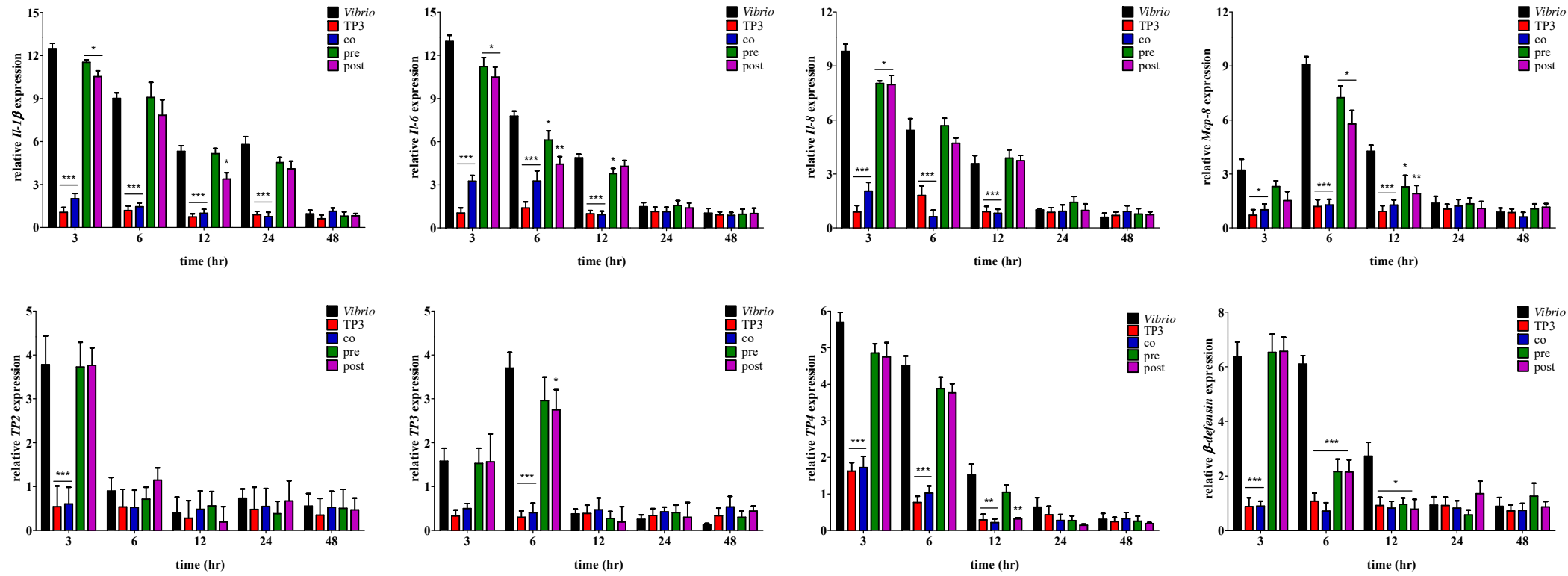

(3c) TP4 treatment of liver

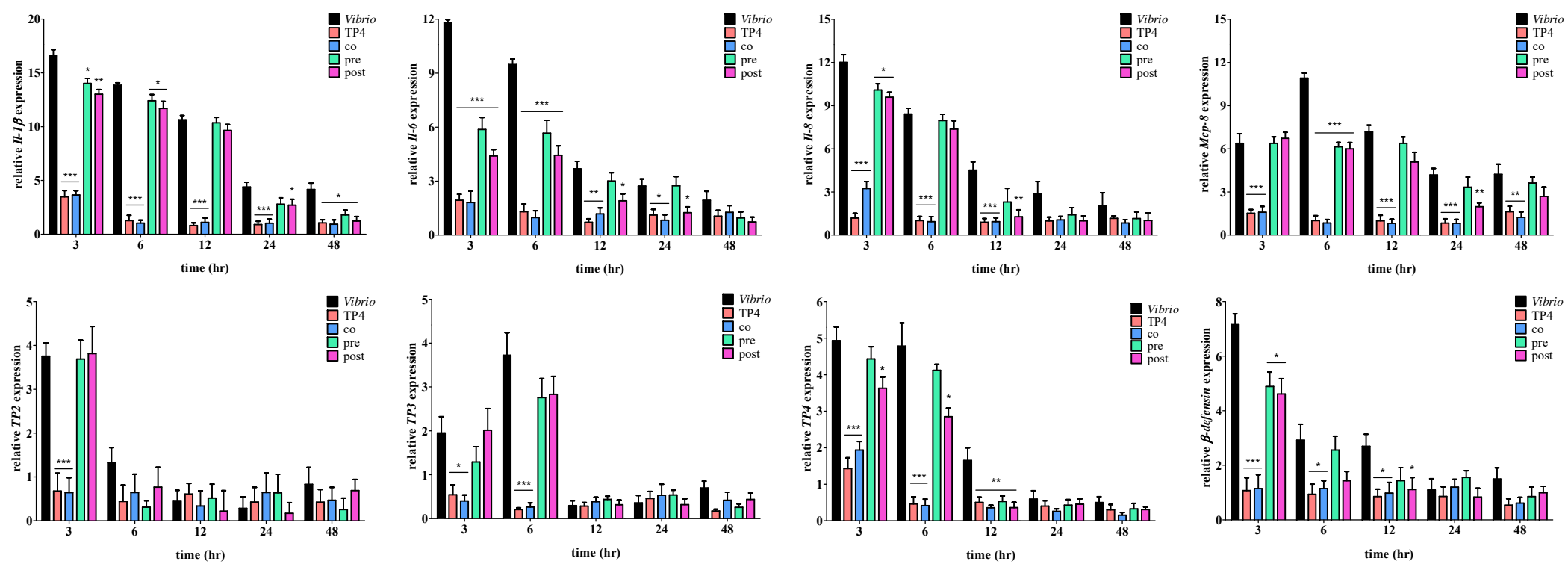

# (3d) TP4 treatment of spleen

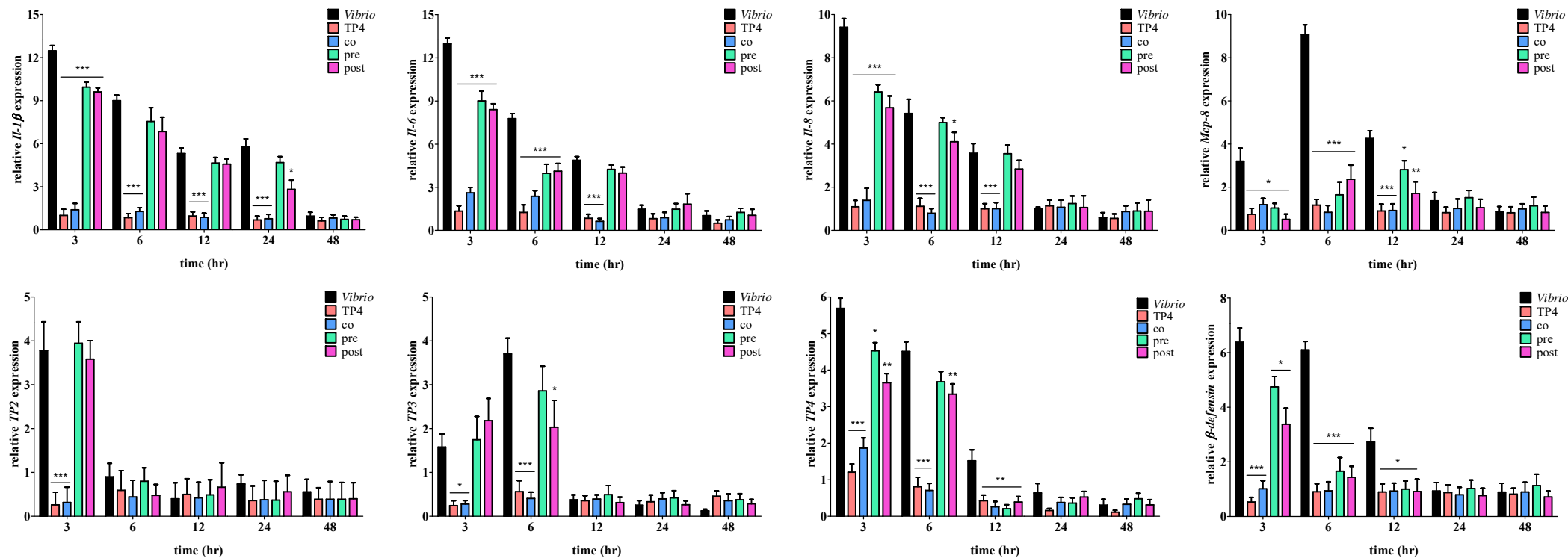

Supplement: S3 Fig — (a) TP3-treated livers, (b) TP3-treated spleens, (c) TP4-treated livers, and (d) TP4-treated spleens. Comparative RT-PCR analysis of the mRNA gene expression levels of immune-related genes. The evaluated genes are shown in Table 1 before and after different experimental conditions, as described in Fig 7. Each bar represents the mean value of three experiments including the SE. The results (mean±SE) marked with asterisks were significantly different (* P<0.05, ** P<0.01, *** P<0.001) among the treatments: Vibrio, injected with V. vulnificus alone; TP4, injected with TP4 alone; TP3, injected with TP3 alone; co, co-treatment with AMP and V. vulnificus; pre, prior treatment with AMP followed by infection with V. vulnificus; post, infection with V. vulnificus followed by treatment with AMP. (PDF) [file pone.0169678.s003.pdf]

(a)

TP3

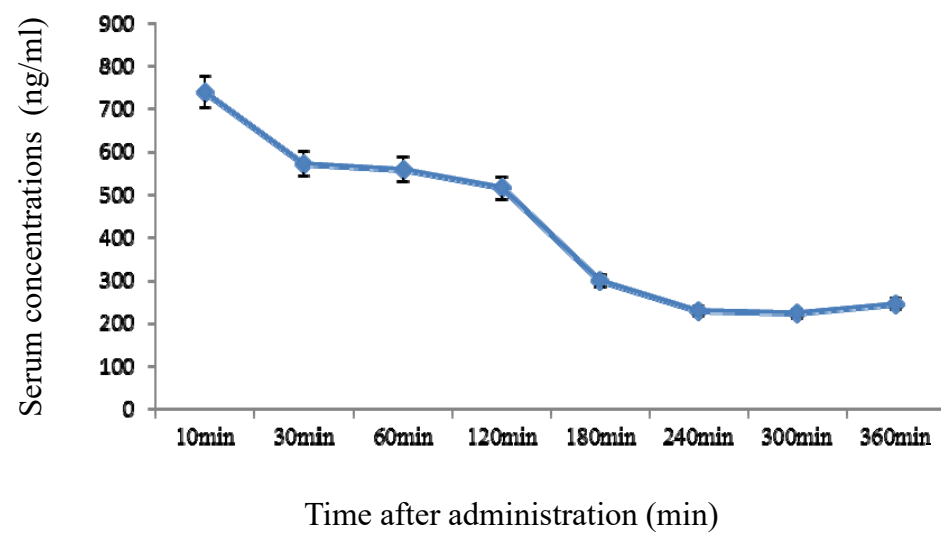

(b)

TP4

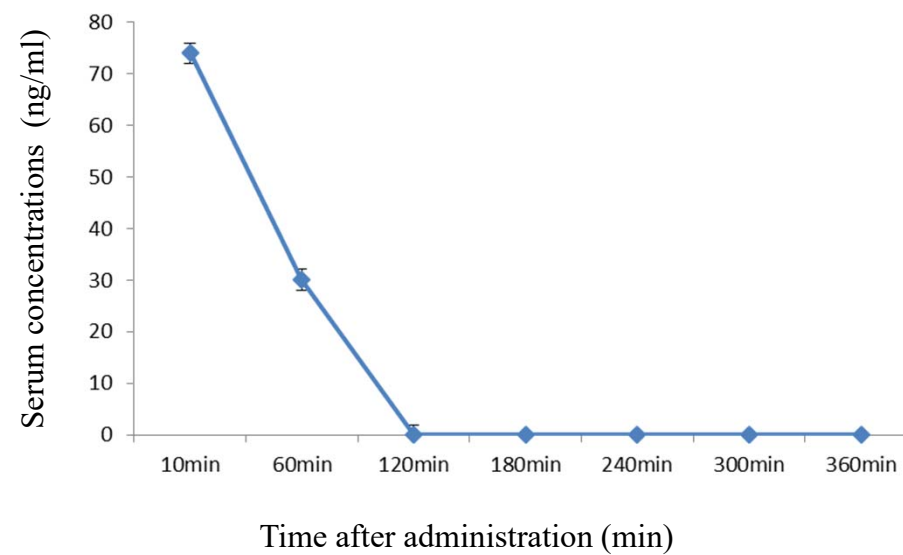

Supplement: S4 Fig — (a) Pharmacokinetic curve for tilapia after injection of a single dose (0.2 mg/kg; using three fish at each time point) of TP3 and (b) TP4. Serum concentrations were determined by intravenous sampling at each time point, and piscidin concentrations were determined by liquid chromatography-mass spectrometry/mass spectrometry (LC-MS/MS). (PDF) [file pone.0169678.s004.pdf]
